# Modular Scanning Confocal Microscope with Digital Image Processing

**DOI:** 10.1371/journal.pone.0166212

**Published:** 2016-11-09

**Authors:** Xianjun Ye, Matthew D. McCluskey

**Affiliations:** Department of Physics and Astronomy, Washington State University, Pullman, WA 99164-2814, United States of America; Universidad Miguel Hernandez de Elche, SPAIN

## Abstract

In conventional confocal microscopy, a physical pinhole is placed at the image plane prior to the detector to limit the observation volume. In this work, we present a modular design of a scanning confocal microscope which uses a CCD camera to replace the physical pinhole for materials science applications. Experimental scans were performed on a microscope resolution target, a semiconductor chip carrier, and a piece of etched silicon wafer. The data collected by the CCD were processed to yield images of the specimen. By selecting effective pixels in the recorded CCD images, a virtual pinhole is created. By analyzing the image moments of the imaging data, a lateral resolution enhancement is achieved by using a 20 × / NA = 0.4 microscope objective at 532 nm laser wavelength.

## Introduction

Confocal microscopy has been used extensively in many areas of science since its invention in 1955 by Marvin Minsky [1]. The ability to obtain optical sections by the aid of a pinhole and detector combination sets it apart from traditional widefield microscopy. Over the last few decades, a variety of confocal microscopes have been developed based on the same principle that a pinhole equivalent blocks out-of-focus light. For example, the use of a single mode fiber emulates the result of the traditional pinhole-photodetector system [2, 3]. A semiconductor laser diode cavity also has the same spatial filtering as a pinhole [4]. With the development of micro electromechanical system (MEMS) technology, digital micromirror device (DMD) based confocal microscopes have gained popularity owing to the fact that DMD can be programmed to create a size-changeable reflection pinhole [5–7]. Image scanning microscopy (ISM), proposed by Sheppard [8], uses pixel reassignment of an array detector, based on the fact that using a displaced point detector has a narrowing effect on the effective point spread function, which improves the lateral resolution of confocal microscopy. ISM was recently experimentally demonstrated by Müller and Enderlein [9]. They used a configuration which places an EMCCD camera behind the confocal pinhole. Another ISM approach uses a specifically-designed point detector array in which each displaced photodiode unit acts individually. Thus, multiple images are acquired with each image representing a different phase of the same sample. These images are then combined using sophisticated algorithms to generate the final sample image, contributing to a resolution improvement in both lateral and axial directions [10].

In this paper, we present a modular scanning confocal microscope in which the pinhole and detector are replaced by a CCD camera [11]. Instead of monitoring the light intensity, the entire beam profile is collected. After data acquisition, the beam profile images are processed to yield a sample image. By eliminating the physical pinhole and using an off-the-shelf CCD camera instead of a custom-designed detector, it reduces system complexity as well as alignment effort. This approach differs from that of Ref [12], which selected active CCD pixels to create a synthetic pinhole. With our microscope, the area of the detector plane is fully analyzed to enhance contrast or determine surface topography. Additionally, our approach provides a way to create multiple pinhole sizes on a single scan. On another note, confocal microscopy has been a mature market in which many commercial microscopes are available, but with a high cost. For this reason, many researchers opt to build custom designed microscopes to balance cost and functionality [13]. Our microscope, which uses low-cost components, could fill an important market need.

## Experiment

**Fig 1:**
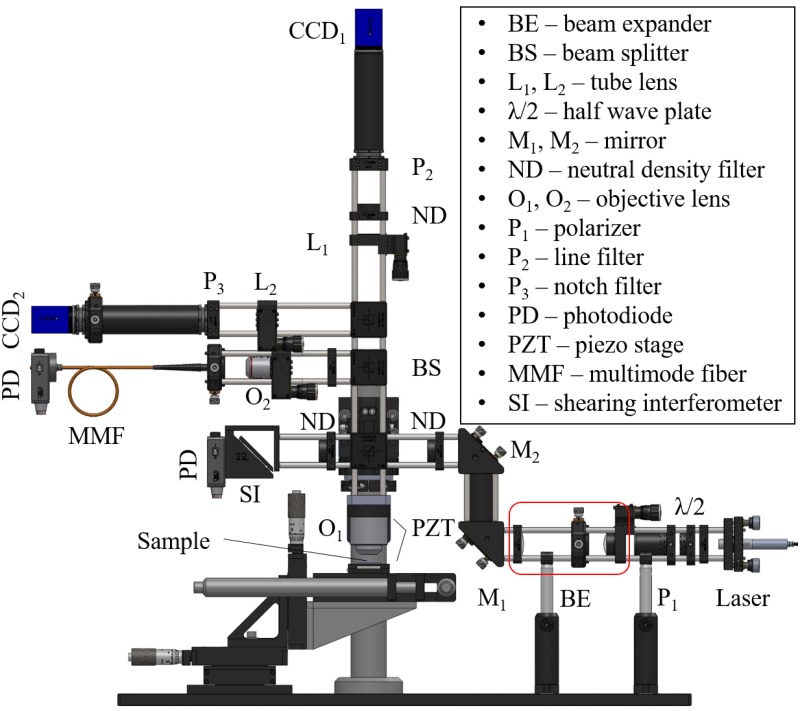
Experimental Setup.

The scanning microscope system as shown in Fig 1 is built around Thorlab’s 30 mm cage system with a few custom designed parts. The entire system consists of four primary modules:

1. The light source module uses a collimated laser diode of 4.5 mW at 532 nm wavelength. A beam attenuation unit made of a Glan-Taylor polarizer P_1_ and a half waveplate is used to adjust the incident laser power. A Keplerian type beam expander expands the laser beam to slightly overfill the back aperture of the microscope objective O_1_ (Zeiss LD Plan-Neofluor 20 × /0.4 Corr). A 50 *μ*m diameter confocal pinhole is inserted at the internal focal point acting as a spatial filter. The expanded beam is then guided into the main microscope optical train by a beam steering mirror pair and a beam splitter cube. The reflected (or emitted) light is then directed by various beam splitter cubes (or dichroic mirrors) into the camera detection module and fiber-optic detection module.
2. The camera module includes two arms. CCD_1_ (The Imaging Source, DMK 23U618 monochrome camera) in the vertical arm collects the reflected light pattern, saved as a 640 × 480 resolution bitmap for later image processing. A line filter at 532 nm is placed in front to only let reflected laser light pass through. CCD_2_ (The Imaging Source, DFK 23U274 color camera) in the horizontal arm is used for wide field inspection and initial positioning, and a notch filter at 532 nm is used to keep laser light from entering the camera. A 200 mm focal length tube lens is used in both arms.
3. The fiber detection module uses a 4 × Olympus microscope objective to couple light into a 25 *μ*m diameter multimode fiber, which acts as the confocal pinhole.
4. The motion module uses a piezoelectric objective scanner (Physik Instrumente, PIFOC^®^ P-725.4CD) for vertical scan, and a piezoelectric nanopositioning stage (Physik Instrumente, P-611.2S) for lateral scan. A motorized scan table (Physik Instrumente, KT-120) and a 3-axis manual stage are used for large area translation.

In addition, a shearing interferometer is used for beam collimation and intensity monitoring. A LabJack U3-LV DAQ device is used for collecting data from the photodiodes. The data (image) acquisition and motion control software is written in the C++ programming language. Image processing and data analysis are done in MATLAB^®^ and Origin^®^.

We choose sample scanning instead of laser scanning because the design is optically simple and less susceptible to optical aberrations. Additionally, the field of view (FOV) of a sample scan microscope can be independently chosen, without changing the microscope’s optical design.

By selecting a region of interest (ROI) on recorded images, a virtual pinhole can be created. The ROI only includes the pixels corresponding to the diffraction limited spot on the sample plane, which abides by the confocal principle. In practice, we choose a square pinhole of N × N pixels in our experiment. The choice of N value will be discussed in the following section. Beyond a virtual pinhole, the image can be analyzed to bring out additional contrast.

Once the scan is complete, the recorded images are stored in the same order as their spatial coordinates. Then computer algorithms are used to extract information from the recorded images.

## Image Processing

### Image Moment

Images are analyzed by calculating image moments. The concept of moments is extensively used in classical mechanics [14]. A general definition of a two-dimensional (p+q)^th^ order moment of a density distribution f(x,y) is given by [15]:

$$\begin{array}{c} M_{\text{pq}}=\int_{-\infty }^{\infty }\int_{-\infty }^{\infty }x^{p}y^{q}f\left(x,y\right)\text{dxdy},p,q=0,1,2,\ldots \end{array}$$ (1)

Its form in image processing is provided as follows:

$$M_{\text{pq}}=\sum_{x}\sum_{y}x^{p}y^{q}I\left(x,y\right)$$ (2)

Thus, the 0^th^ order image moment M_00_ is equivalent to the irradiance. As discussed in the following section, higher-order moments yield novel contrast.

### Two-dimensional Scan

To illustrate our microscope’s operation and its performance, we started with a two-dimensional scan of a 100 *μ*m × 100 *μ*m area on a 3 mm × 3 mm chip carrier sample with total scan points of 200 × 200 at a 0.5 *μ*m step size along both X and Y. Final images are obtained by summing the 0^th^ order image moment M_00_ over a ROI of N × N pixels on CCD_1_ recorded images using Eq 2. The Nyquist-Shannon sampling theorem requires at least two pixels per resolvable element. We choose a square pinhole of 2 × 2 pixels as the smallest to start with. Additional cropping sizes (N = 6, 11, 21, 31, 41, 51, 101) are used to determine the optimal pinhole size.

As a comparison, we used the fiber-optic module to represent the standard confocal signal detection. A 25 *μ*m diameter multimode fiber was used to collect reflected light from the same field of view.

**Fig 2:**
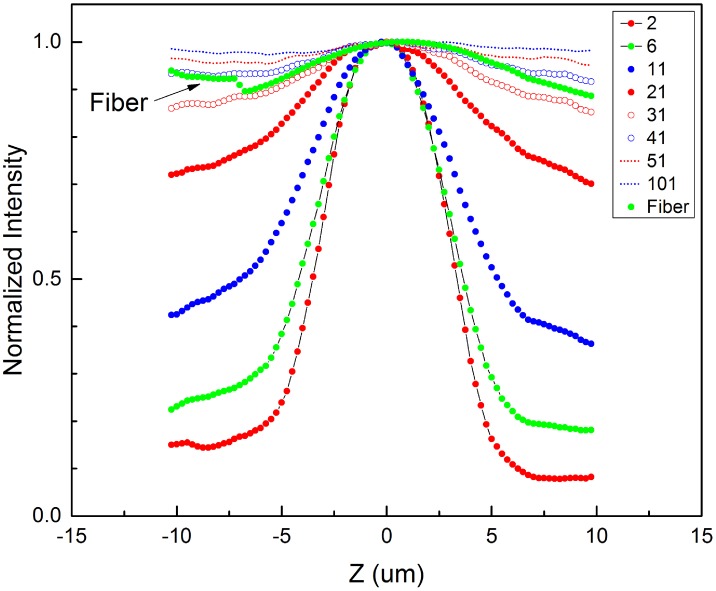
Axial response curves for fiber-optic and CCD based detection.

Before running an actual sample scan, a calibration process was carried out to ensure that both the fiber facet and the CCD are in a conjugate plane to the focal plane of the objective lens. Fig 2 shows the measured axial response curves of the fiber-optic detection and the CCD based detection. These relatively symmetrical plots share a common axial zero/focal point suggesting that they are optically aligned to the same conjugate plane. The fiber-optic response curve is relatively broader than those in the CCD plot up to 31 × 31. In the CCD plot, the width of the curve increases with the crop size. The full width at half maximum (FWHM) is 7 *μ*m for N = 2, and 25.72 *μ*m for N = 21. Beyond 31 × 31, a well-defined peak is not found.

**Fig 3:**
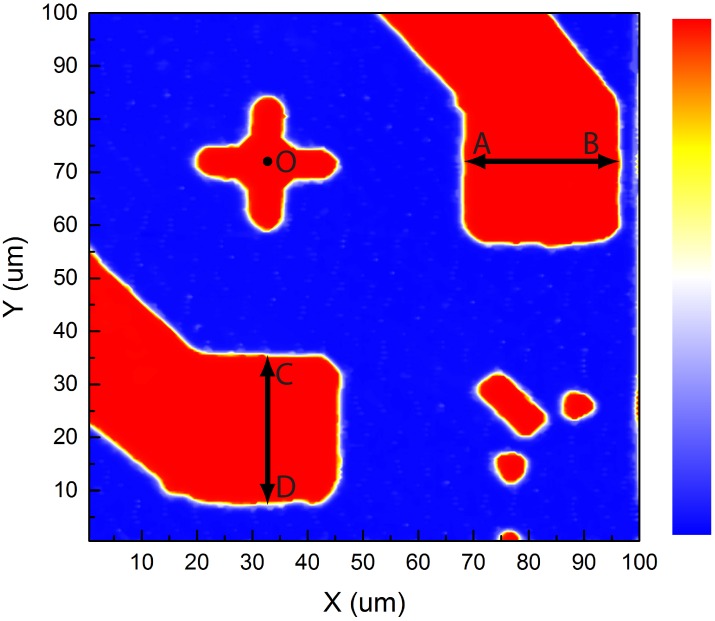
Image of a chip carrier sample by fiber-optic confocal scan. A 100 *μ*m × 100 *μ*m area on a 3 mm × 3 mm chip carrier, obtained by plotting the acquired intensity map from fiber-optic detection.

**Fig 4:**
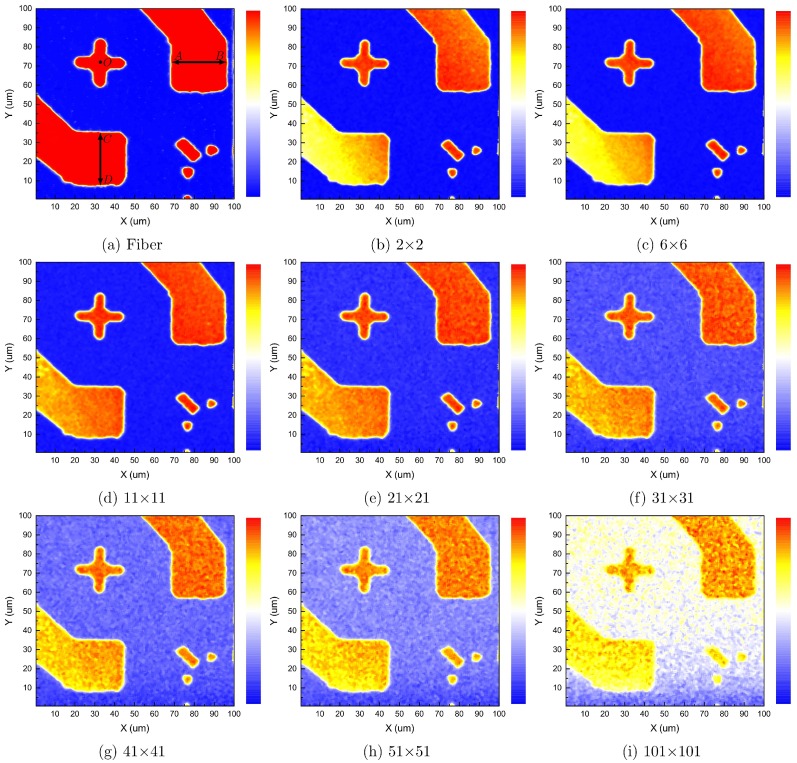
Images of a chip carrier sample by CCD based confocal scan. A 100 *μ*m × 100 *μ*m area on a 3 mm × 3 mm chip carrier, obtained by analyzing a N × N pixel ROI on CCD_1_ recorded images. Gold electrodes are at the bottom-left and top-right. The plus sign is polarity marker. Bottom-right are gold position markers.

An intensity map revealing the geometric profile of the sample from the fiber-optic confocal scan is shown in Fig 3. The CCD based detection results are shown in Fig 4.

**Fig 5:**
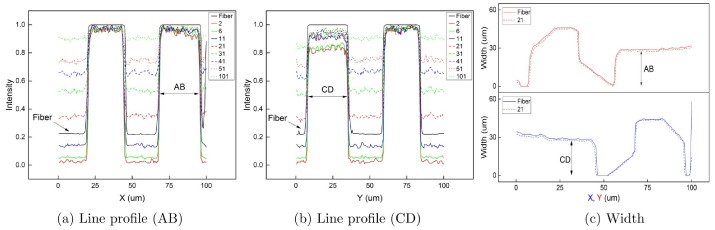
Intensity profiles and width profiles. (a) Intensity profile of a horizontal line passing through O shows the horizontal width of the polarity marker and the electrode (AB) of different pinhole sizes at a specific Y position. (b) Intensity profile of a vertical line passing through O shows the vertical width of the electrode (CD) and the polarity marker of different pinhole sizes at a specific X position. (c) Full width profiles of electrodes across entire X and Y ranges measured in fiber-optic case and CCD case with a pinhole size of 21 × 21 pixels.

**Fig 6:**
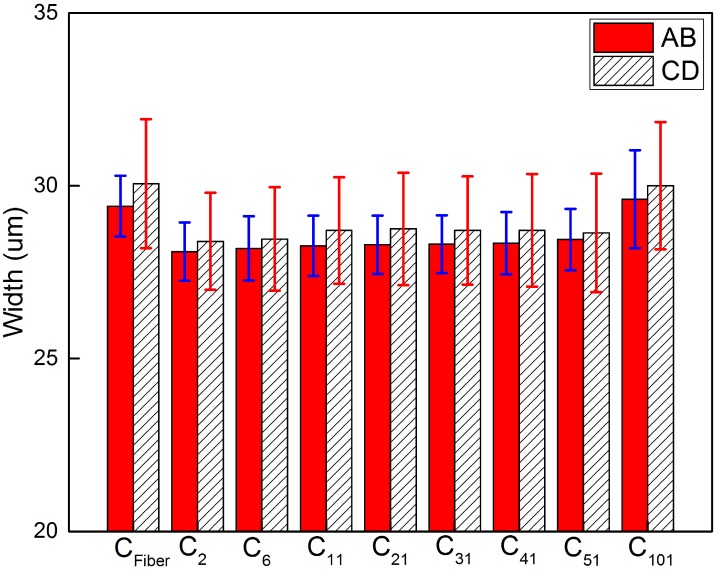
Electrode width measurement.

By manual control of the PI-611.2S piezoelectric stage to translate across the width of the top-right and bottom-left electrodes, a visual estimate of the electrode width AB and CD were obtained when laser spot vanished at the opposite edges in the inspection camera CCD_2_, i.e., AB, 28-29 *μ*m, CD, 27-30 *μ*m. In a more objective approach, a computer program was written to calculate the average width of the top-right and bottom-left electrodes, AB and CD, instead of a discrete set of hand-picked line pairs. It first calculates a threshold to completely separate the gold plated electrodes and markers from the substrate, and then computes the width of the electrodes above this threshold at each X or Y position. Thus, an array of width values is obtained. Fig 5(c) shows the width profiles. The upper half of Fig 5(c) represents maximum horizontal width of electrodes at each Y position. The lower half of Fig 5(c) represents maximum vertical width of electrodes at each X position. The average width of this array of data and their standard deviations are shown in Table 1, and plotted in Fig 6. Intensity profiles of horizontal and vertical lines passing through the center of the polarity marker (O) in Fig 5(a) and 5(b) show a decrease of contrast and an increase of noise with the increase of pinhole size.

**Table 1:** Electrode width measurement.

	AB(*μ*m)	CD(*μ*m)	*σ*_AB_(*μ*m)	*σ*_CD_(*μ*m)
C_Fiber_	29.41	30.06	0.88	1.87
C_2_	28.10	28.39	0.84	1.40
C_6_	28.19	28.46	0.93	1.49
C_11_	28.26	28.71	0.87	1.54
C_21_	28.29	28.75	0.84	1.62
C_31_	28.31	28.71	0.84	1.56
C_41_	28.34	28.71	0.90	1.63
C_51_	28.45	28.63	0.89	1.71
C_101_	29.61	30	1.42	1.84

The diffraction limited spot diameter in the focal plane of the objective lens (Zeiss LD Plan-Neofluor 20 × /0.4 Corr) is 1.62 *μ*m at 532 nm. It is projected by the microscope optics onto an area of diameter 39.56 *μ*m on the CCD, which is enclosed by 7 × 7 pixels. The projected area on the fiber facet in the fiber-optic detection arm is of diameter 8.9 *μ*m. Thus the 25 *μ*m diameter multimode fiber core encloses 2.81 × Airy units (AU), which corresponds to an equivalent area of 20 × 20 pixels on the CCD.

Case 2 × 2, which is at the size limit of point detection by a CCD, shows the least amount of deviations in the width measurement. Larger pinhole sizes, 6 × 6 and 11 × 11, shows slightly larger width values, but maintains comparable standard deviation values to 2 × 2 case. Case 21 × 21 shows slight noise increase. Above 31 × 31, the noise level increases drastically, and the resolution decreases as seen from the fact that the edge profiles of the bottom-right gold position markers become more and more rounded. Case 101 × 101, which corresponds to wide-field imaging, shows the largest standard deviations and the highest noise level. The increase of noise in the larger pinhole cases can be attributed to the inclusion of more non-photon-receiving pixels subject to read noise and dark current noise outside the diffraction limited spot, which increases the overall noise level of the reconstructed image. The fiber-optic case shows larger numerical values due to its moderate core diameter size. In theory, an infinitesimal pinhole could give the best spatial resolution, but that small pinhole also rejects photons which could have been used to counter the noise. Case 6 × 6 gives the closest match to of the 2 × 2 case, and still maintains a sizeable amount of pixels. Thus we choose 6 × 6 as the optimal pinhole size for three-dimensional scans which require more complex analysis and the best performance, but 21 × 21 as the pinhole size for two-dimensional scans which requires a meaningful comparison between fiber and CCD experiments and less computational overhead.

In our experimental configuration, the total scan time was found to only depend on the detectors’ readout speed. The CCD and the photodiode were configured to read out sequentially within each programming loop. A high-precision timing routine determined that the readout time for each point was 18.5 ms (740 seconds in total), i.e., 16.7 ms on CCD readout (60 fps) and 1.8 ms on photodiode readout. Since the photodiode’s rise time is on the order of 1 ns, the 1.8 ms was the single point readout time of the LabJack U3-LV DAQ device through which the photodiode was connected to the computer.

### Lateral Resolution Test

**Fig 7:**
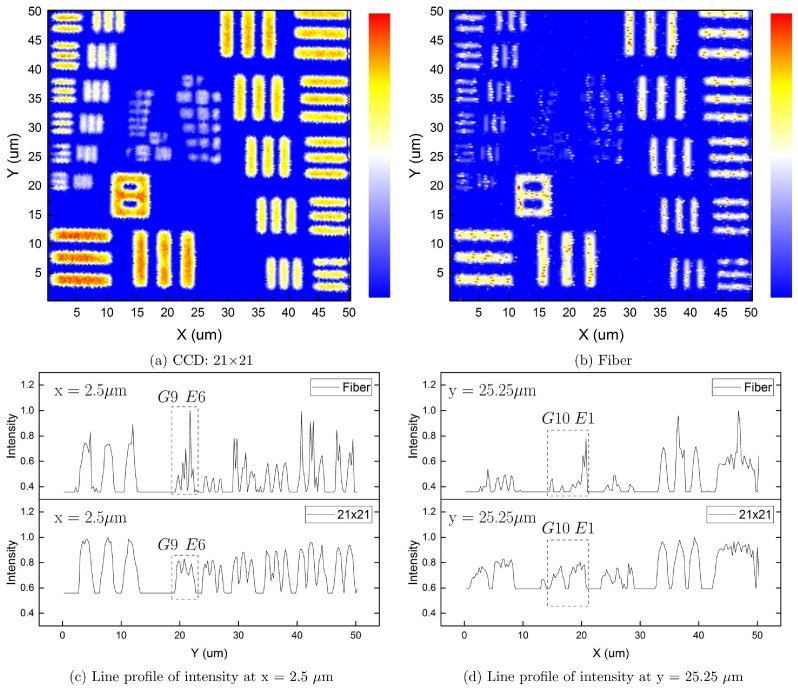
Images of a USAF resolution target. A 50 *μ*m × 50 *μ*m area on a USAF resolution target, obtained in CCD and fiber experiments. (a) Image from CCD case with a pinhole size of 21 × 21. (b) Image from fiber-optic case. (c) Line profile of intensity at x = 2.5 *μ*m. (d) Line profile of intensity at y = 25.25 *μ*m.

To better understand our microscope’s performance, we ran a two-dimensional scan of a USAF resolution target (Ready Optics, California, up to Group 11, Element 6). Fig 7 shows a scan area of 50 *μ*m × 50 *μ*m at a 0.25 *μ*m step size along both X and Y. The CCD experiment uses a 21 × 21 pinhole size which is equivalent to the fiber facet area. Fig 7(c) and 7(d) show the intensity profile along vertical line, x = 2.5 *μ*m, and horizontal line, y = 25.25 *μ*m. The CCD experiment resolves the horizontal stripes up to Group 9, Element 6 (line width of 0.548 *μ*m), and vertical stripes up to Group 10, Element 1 (line width of 0.488 *μ*m), which is comparable to the fiber experiment.

### Higher Order Moments

**Fig 8:**
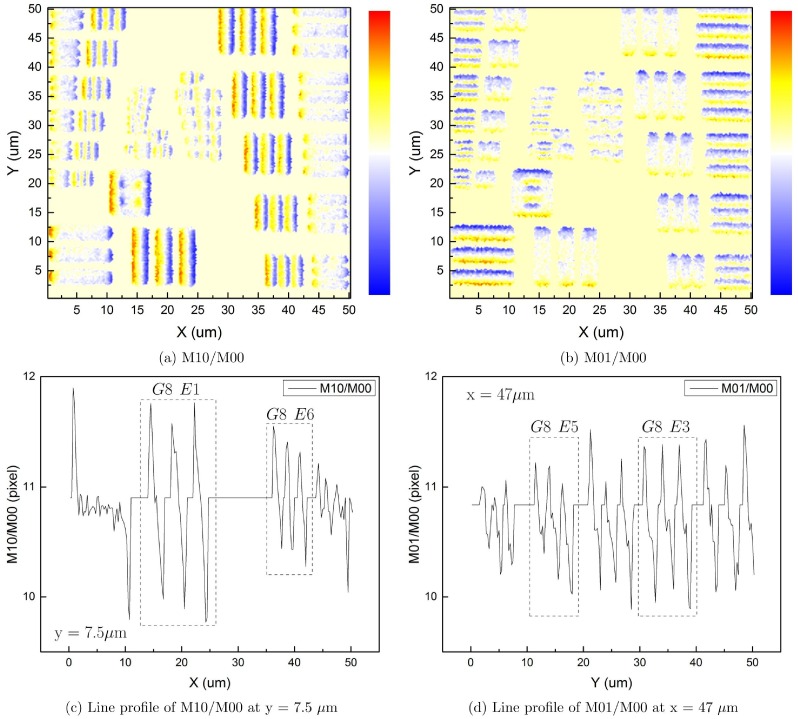
Higher order image moments analysis. (a) M_10_/M_00_ plot shows the shift of the laser spot’s centroid in X direction. (b) M_01_/M_00_ plot shows the shift of the laser spot’s centroid in Y direction. (c) Line profile of M_10_/M_00_ at y = 7.5 *μ*m. (d) Line profile of M_01_/M_00_ at y = 47 *μ*m.

In addition to the 0^th^ order image moment, higher order image moments are also used. Fig 8 shows the results of M_10_ and M_01_ normalized by M_00_ for a 21 × 21 pixel pinhole size. These two quantities represent the (x, y) coordinate of the centroid of the image:

$$\begin{array}{c} \langle x\rangle =\frac{M_{10}}{M_{00}}\;,\;\langle y\rangle =\frac{M_{01}}{M_{00}} \end{array}$$ (3)

The patterns shown in Fig 8(a) and 8(b) resemble the behavior of an edge filter. By looking at the color of stripe patterns, we found that the centroid show an inward-moving trend on the edge of stripes at the laser intensity level used in this experiment. More specifically, 〈*x*〉 increases on the left edge, and decreases on the right edge. 〈*y*〉 increases on the lower edge, and decreases on the upper edge. By carefully analyzing the line profile of intensity data, we found the spacing between peaks in Fig 8(c) and (d) match the designed width of a line-pair closely. Fig 8(c) gives a line width of 1.91 ± 0.25 *μ*m comparable to that of Group 8, Element 1 (line width of 1.953 *μ*m), and a line width of 1.13 ± 0.25 *μ*m comparable to that of Group 8, Element 6 (line width of 1.096 *μ*m). Fig 8(d) gives a line width of 1.56 ± 0.25 *μ*m comparable to that of Group 8, Element 3 (line width of 1.550 *μ*m), and a line width of 1.22 ± 0.25 *μ*m comparable to that of Group 8, Element 5 (line width of 1.230 *μ*m). This means, a higher order image moment analysis of the laser spot can reveal the slight shift of the centroid distribution, and provide a precise and straightforward determination of the dimensions of the pattern of interest without the need for a threshold. This method provides a view of how laser spot’s internal intensity distribution changes when the laser overlaps a sharp edge.

**Fig 9:**
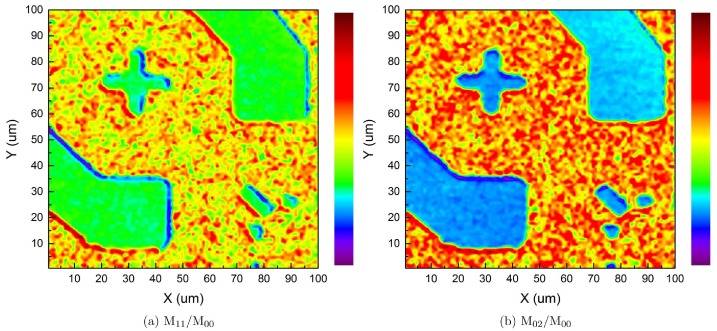
Higher order image moment analysis of electrode surface. (a) M_11_/M_00_. (b) (a) M_02_/M_00_.

Fig 9 shows another example of higher order moment application. In this case, extra details of the feature on the electrode surface are brought out, compared to the 0^th^ order image moment case and the fiber-optic confocal case, and the edge profiles are more refined. Overall, image-moment analysis leads to an enhancement of the reconstructed image.

### Image Differences

**Fig 10:**
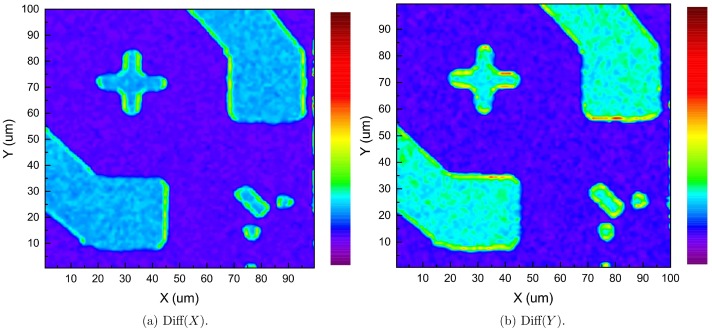
Image differences between adjacent points used to generate edge details.

Besides the above mentioned techniques, by calculating the difference between recorded images of adjacent points on the sample, a more detailed plot of the edges can be obtained. Fig 10 shows the result by doing subtraction of images from adjacent points along X and Y directions. An orientation-dependent pattern is obtained, which can be used to refine the boundaries of the acquired image by aforementioned image moment method.

The algorithm to compute the difference between points (m+1, n) and (m, n) along the X direction is given by

$$\begin{array}{c} {\text{Diff}}_{m,n}\left(X\right)=\text{sqrt}\left(\sum_{i}^{N}\sum_{j}^{N}{\left(I_{m+1}\left(i,j\right)-I_{m}\left(i,j\right)\right)}^{2}\right) \end{array}$$ (4)

likewise, the difference between points (m, n+1) and (m, n) along Y direction is given by

$$\begin{array}{c} {\text{Diff}}_{m,n}\left(Y\right)=\text{sqrt}\left(\sum_{i}^{N}\sum_{j}^{N}{\left(I_{n+1}\left(i,j\right)-I_{n}\left(i,j\right)\right)}^{2}\right) \end{array}$$ (5)

where N × N is the size of the image (in this case, N = 6).

### Three-dimensional Scan

**Fig 11:**
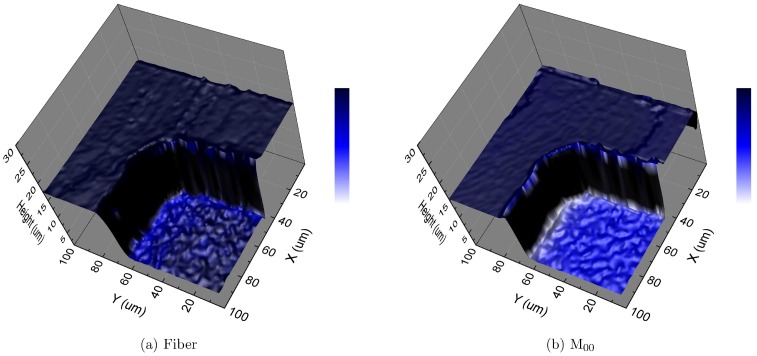
3D depth measurement of an etched silicon wafer sample.

In order to evaluate the three dimensional scan performance, a piece of photolithography-etched silicon wafer was tested. A total volume of 100 *μ*m × 100 *μ*m × 30 *μ*m was scanned with a step size of 1 *μ*m for all XYZ on this geometrically simple object. Fig 11 shows the reconstructed sample images of the fiber-optic confocal scan and the CCD based confocal scan. A pinhole of 6 × 6 pixels was used in the CCD scan for optimal result. The height value for each XY coordinate was determined by the location of maximum intensity or maximum image moment M_00_ along the Z direction. Then a topographic surface was reconstructed using these height values. Both images show that the etched surface is lower than the un-etched surface by a certain depth value, and is also grainier.

**Fig 12:**
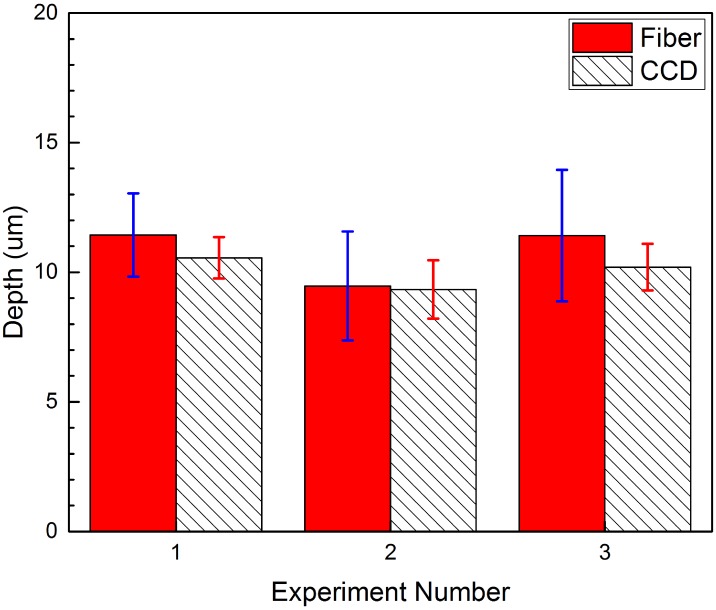
3D depth measurement of an etched silicon wafer sample.

To determine this depth value, a computer program first computes a threshold value to completely separate high ground and low ground, and then calculates the average height value for each of the two surfaces and their standard deviations. The final average depth is the subtraction of the two height values, and the sum of the two standard deviations gives the combined standard deviation. The numerical results of three scans are shown in Table 2 and in Fig 12. Each scan took about 1 hour and 30 minutes, or 18.5 ms per point. Overall, the CCD based scan gives result in agreement with the fiber-optic confocal scan within experimental uncertainty. The CCD scan generates a final image that is smoother than the fiber-optic confocal scan.

**Table 2:** 3D depth measurement of an etched silicon wafer sample.

	Depth_Fiber_(*μ*m)	Depth_CCD_(*μ*m)	*σ*_Fiber_(*μ*m)	*σ*_CCD_(*μ*m)
E_1_	11.43	10.56	1.60	0.80
E_2_	9.47	9.33	2.10	1.12
E_3_	11.41	10.20	2.54	0.90

## Summary

We have demonstrated a modular scanning confocal microscope using a CCD camera and image processing. It has been used to map out a two dimensional surface of a chip carrier and a three dimensional depth profile of an etched silicon wafer. The obtained dimensional information was compared with that acquired by a fiber-optic confocal microscope module at the same time. The performance of this CCD and image processing based approach is on par with the fiber-optic module. However, the CCD has the advantage that its images can be analyzed in a variety of ways to enhance contrast. Additionally, a CCD does not require the precise alignment needed for an optical fiber or pinhole. In this work, we used a off-the-shelf monochrome industrial CCD camera which has a sensitivity of 0.015 lux. For low photon budget experiment, e.g., fluorescence measurement, choices of more light-sensitive scientific camera are available in current market, such as EMCCD and sCMOS. In conclusion, our work provides an easy to use, fully expandable as well as affordable approach to custom build a scanning confocal microscope based on postproccesing image data. Future work includes adding spectroscopic imaging module, providing scanning photoluminescence measurement capability.
